# Prevalence of respiratory syncytial virus infection and associated factors in children aged under five years with severe acute respiratory illness and influenza-like illness in Ethiopia

**DOI:** 10.1016/j.ijregi.2024.01.004

**Published:** 2024-01-17

**Authors:** Adamu Tayachew, Gizaw Teka, Ayele Gebeyehu, Wolde Shure, Mengistu Biru, Leuleseged Chekol, Tsegaye Berkessa, Eyasu Tigabu, Lehageru Gizachew, Admikew Agune, Melaku Gonta, Aster Hailemariam, Ergetu Gedefaw, Adane Woldeab, Ayinalem Alemu, Yimam Getaneh, Leuel Lisanwork, Kalkidan Yibeltal, Ebba Abate, Aschalew Abayneh, Mesfin Wossen, Mesay Hailu, Firehiwot Workineh

**Affiliations:** 1 Ethiopian Public Health Institute, Addis Ababa, Ethiopia; 2 The Ohio State University Global One Health, Addis Ababa, Ethiopia; 3 Addis Continental Institute of Public Health, Addis Ababa

**Keywords:** Epidemiology, RSV, SARI/ILI, Surveillance, Ethiopia

## Abstract

**Objectives:**

Acute respiratory infections because of respiratory syncytial viruses (RSVs) are among the major leading causes of morbidity and mortality in children worldwide. RSV prevalence and its contributing factors among children aged under 5 years in Ethiopia are not well studied. To assess the prevalence and associated factors of RSV infection in children aged under 5 years using influenza sentinel surveillance sites in Ethiopia.

**Methods:**

A cross-sectional study design was used utilizing influenza-like illness/sever acute respiratory illness surveillance data from January 2021 to December 2022 at the Ethiopian Public Health Institute.

**Results:**

In total, 2234 cases were included, with an overall RSV positivity rate of 16.2%. The RSV positivity rate was high in children aged under 1 year (22.8%) and during fall season (24.8%). The RSV positivity rate was significantly associated with ages under 1 year (adjusted odds ratio [AOR] 2.8, 95% confidence interval [CI]: 1.89-4.15) and 1-2 years (AOR 1.9, 95% CI: 1.26-2.73) and the fall season (AOR 1.67, 95% CI: 1.17-2.38).

**Conclusion:**

The study revealed that a considerably high RSV positivity rate was detected in children aged under 5 years. The age of children and season have a significant association with RSV positivity rate. Further studies of RSV viral genotype, clinical characteristics, and disease outcome need to be conducted for a better understanding of the virus and disease outcome.

## Abstract (author highlights)

Highlights

• Influenza-like illness/severe acute respiratory illness surveillance can be leveraged to encompass viruses including respiratory syncytial virus (RSV).
• RSV found in 16.2% of children aged under 5 years with influenza-like illness and sever acute respiratory illness in Ethiopia.
• Younger aged children are more affected by RSV than the older ones.
• RSV infections are significantly associated with age of children and season.

## Introduction

Acute respiratory tract infection (ARTI) is one of the major health issues in infants and children in the world causing morbidity and mortality and it is the most common reason for consultation and hospitalization in children and adults [1]. The respiratory syncytial virus (RSV) was first isolated in 1955 from chimpanzees with a respiratory illness at the Walter Reed Army Institute of Research in the United States. Later on in 1960, the virus was also detected in infants with severe lower respiratory illness [2].

Worldwide, the RSV is responsible for about 60,000 annual in-hospital deaths in children under 5 years and a leading viral cause of severe respiratory disease and hospitalization in young children [3]. Approximately 45% of the hospitalizations and deaths are caused by RSV-associated acute respiratory tract infection in infants aged under 6 months. In 2015 alone, 33.1 million new episodes of RSV-associated ALRI occurred worldwide in children aged less than 5 years, with at least 3.2 million hospitalizations and 59,600 in-hospital deaths [4].

The RSV results in clinical syndromes that include upper and lower respiratory tract disease [5]. Moreover, respiratory viral infections, including RSV infections, increase the risk of secondary bacterial infections, pneumonia, and sepsis [6]. The RSV virus is highly contagious transmitted by aerosol, contact with contaminated surface, and direct contact with infected individuals. Moreover, the occurrence of RSV transmission has seasonal patterns and as in many African countries of the tropics, such as Madagascar, Burkina Faso, and Mozambique, it occurs year-round and peaks during rainy seasons, although this may not true across other countries of African regions [7].

Most RSV infections present with nonspecific symptoms, and a definitive diagnosis for RSV primarily depends on laboratory investigation [2]. Currently, there are no effective treatment or vaccine available for RSV, except the Arexvy vaccine, which is manufactured by Fizzer Pfizer and recently approved by US Food and Drug Administration to be used for elderly people aged >60 years [8]. The RSV immunoprophylaxis called palivizumab is also approved for use in high-risk pediatric populations; thus, the reduction of morbidity and mortality in the general population is mainly relied on preventive measures [2].

Study reports from different parts of the world showed different RSV test positivity rates. A systematic review report conducted in Europe, Asia, and other regions in 2015 in those aged under 5 years with community-acquired pneumonia showed a 17.5% RSV positivity [9], whereas a 24.4% RSV positivity was reported in the Middle East and North Africa in 2020 [10]. A study of 2363 study participants from Malawi by Peterson et al. [11] showed an RSV positivity of 11.9% and had a strong association with severe acute respiratory illness (SARI) cases with warning signs. A surveillance data report from 2009-2012 from Kenya showed positivity rates of 11.9% and 10.4% for SARI and influenza-like illness (ILI) cases of those aged under 5 years, respectively [12].

Acute respiratory tract infections are among the major public health problems in Ethiopia. In the 2019 health and health-related indicator report by the Ministry of Health, Ethiopia showed that upper respiratory tract infections and pneumonia are among the top five causes of morbidity and mortality, respectively [13]. In Ethiopia, most acute respiratory illness (ARI) cases are treated empirically and overuse and inappropriate use of antibiotics for the relief of ARI is common [14].

There are no sufficient data showing the prevalence and associated factors of RSV infection in children under 5 years of age with acute respiratory infections in Ethiopia. As one of the major contributors for the acute respiratory infection–related morbidity and mortality, the RSV infection prevalence and associated factors need to be investigated and analyzed to better support the prevention and case management process. Thus, this study aimed to assess the prevalence and associated factors of RSV infection in children under 5 years with ILI and SARI in Ethiopia.

## Methods

### Study setting

In Ethiopia, the Ethiopian Public Health Institute started the influenza sentinel surveillance in 2008 using two sentinel surveillance sites: one ILI and one SARI site located in Addis Ababa, the capital city. The sentinel surveillance was then expanded to include additional sites in Addis Ababa and across regions of Ethiopia. Currently, there are 21 influenza sentinel surveillance sites in the country, which are designated as ILI (all in Addis Ababa) and SARI sites, distributed across Addis Ababa and regional administrative states. Furthermore, four regional influenza and other respiratory virus molecular testing laboratories were established in Addis Ababa, Oromia, Amhara, and Southern Nation, Nationalities, and Peoples Region in August 2022 to build regional molecular testing capacities and decentralize the ILI/SARI testing system, which, in turn, can strengthen the surveillance and outbreak detection system.

**Figure 1:**
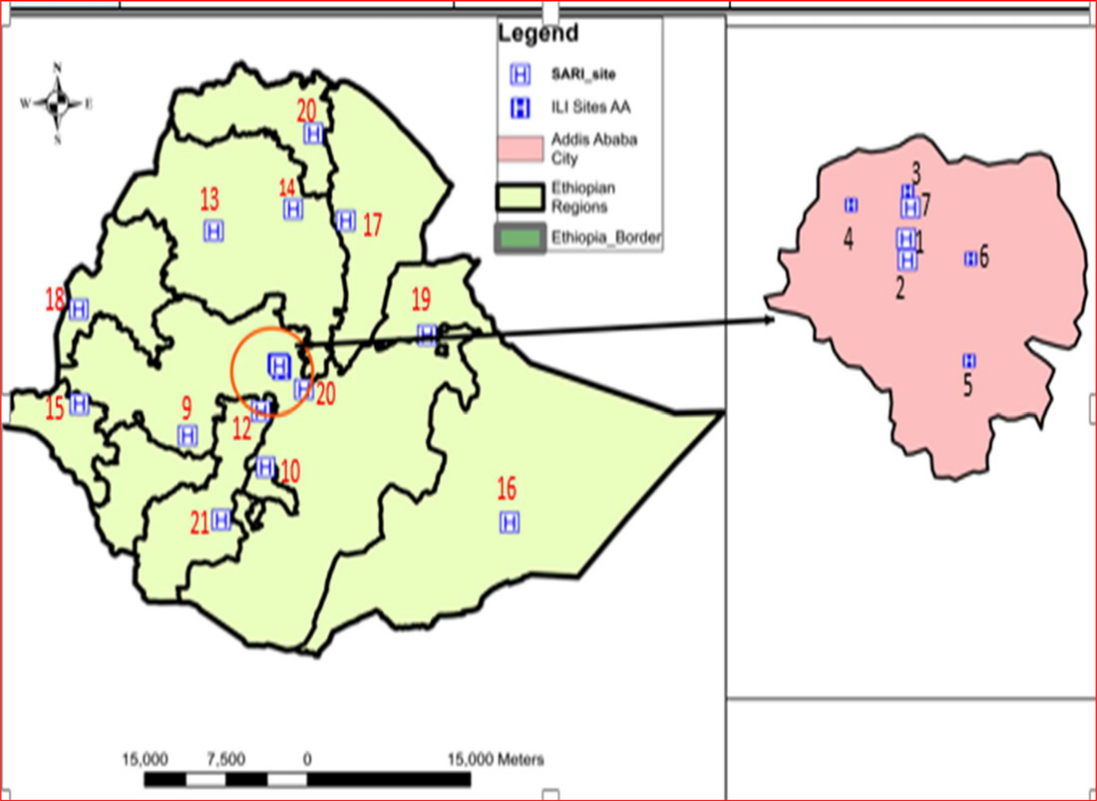
Distribution of study sites across the regions and Addis Ababa. The numbers indicated from the map are the name of the facility as listed in Supplemental Table 1. ILI, influenza like illness; SARI, severe acute respiratory illness.

This study included data from 19 actively working facilities and excluded two sites. There were no surveillance data received from sites in Mekele, Tigray owing to security issues and from Jinka, Southern Nation, Nationalities, and Peoples Region because the site was not operation in 2021-2022 (Figure 1, Supplemental Table 1).

### Data collection tools, procedure, and data quality assurance procedure

Data collected form the influenza sentinel surveillance system were used to conduct the RSV prevalence study. The data were collected by trained nurses, surveillance experts, laboratory technicians, or doctors at each sentinel sites. The sentinel sites used uniform and standardized case reporting formats adopted from the World Health Organization for ILI and SARI sentinel surveillance purposes. This standardized case investigation format consists of laboratory, clinical, and epidemiological information of each case. The samples from the sentinel sites and regional laboratories were transported to the testing laboratories on weekly and biweekly schedules. The laboratory testing for RSV was done by reverse transcription-polymerase chain reaction (RT-PCR), along with influenza and SARS-CoV-2, according to the kit manufacturer's standard operating procedure instructions.

### Data analysis

All the demographic and test result data from Excel were coded and was exported to IBM SPSS version 20 for statistical analysis. Descriptive summary measures, such as frequencies and proportions, were computed to characterize the socio-demographic and clinical characteristics with the RSV test results. The association of each independent variable with the dependent variable was assessed using the binary logistic regression. The odds ratio (OR) along with the 95% confidence interval [CI] was used to assess the strength of association between the RSV positivity rate and the independent risk factors. Variables with *P*-value below 0.05 in the multivariable analysis were considered to have a statistically significant association.

## Results

### Demographic profile of the study participants

The total number of children under 5 years of age with completed RSV polymerase chain reaction test result enrolled by the ILI/SARI surveillance system during 2021-2022 were 2234 and of these, 1311 (59%) were males. The majority of the study participants were below 2 years of age (63%) and the average age of study participants was 1 year and 4 months and had an SD of 1.22. Moreover, the majority of study participants were SARI cases (1919 [86%]). About half of the study participants were residents of the capital city, Addis Ababa (Table 1).

**Table 1:** Demographic description of the study participants.

Variable	Category	Number of cases	Percentage (%)
Sex	Female	923	41.3
	Male	1311	58.7
Age (in years)	<1	604	27.0
	1-2	848	38.0
	2-3	396	17.7
	3-5	386	17.3
Case classification	Influenza like illness (Outpatient)	315	14.1
	Sever acute respiratory illness (Inpatient)	1919	85.9
Region	Addis Ababa	1072	48.0
	Other regions	1162	52.0
Influenza test result	Positive	179	8.0
	Negative	2055	92.0
SARS-CoV-2	Positive	74	3.3
	Negative	2160	96.7
Year	2021	332	14.9
	2022	1902	85.1
Season	Winter	742	33.2
	Spring	641	28.7
	Summer	396	17.7
	Autumn	455	20.4

### RSV positivity rate

Of all the 2234 tested children, 362 (16.2%) were positive for RSV by RT-PCR. RSV positivity rates of 15.9% in male and 16.7% in female study participants were detected. The RSV was detected across all age groups, with the highest positivity rate (22.8%) in infants and the lowest positivity rate (9.8%) in children older than 3 years of age. The RSV positivity in children with ILI was 15.2% (48), whereas the RSV positivity rate in children with SARI was 16.4% (314). A fairly higher positivity rate (17.2%) was also detected in children living in Addis Ababa compared with the 15.3% positivity rate outside of Addis Ababa (Table 2).

**Table 2:** Factors association with respiratory syncytial virus infection among under 5 children with severe acute respiratory and influenza like illness, 2021-2022, Ethiopia.

Variable		Respiratory syncytial virus polymerase chain reaction result		COR (95% confidence interval)	*P*-value	AOR	*P*-Value
		Positive (%)	Negative (%)				
Sex	Female	154(16.7)	768(83.3)	1.06(.85-1.33)	0.60		
	Male	208(15.9)	1103(84.1)	1.0		1.0	
Age (in years)	<1	138(22.8)	466(77.2)	2.712(1.85-3.99	<.001^a^	2.53(1.71-3.73)	<0.001^b^
	1-<2	140(16.5)	708(83.5)	1.811(1.24-2.65)	0.002^a^	1.78(1.21-2.61)	0.003^b^
	2-<3	46(11.6)	350(88.4)	1.204(.76-1.90)	0.424	1.18(.75-1.87)	0.469
	3<-=5	38(9.8)	348(90.2)	1.0		1.0	
Case classification	Influenza like illness (outpatient)	48(15.2)	267(84.8)	1.0		1.0	
	Severe acute respiratory illness (outpatient)	314(16.4)	1604(83.6)	1.098(.79-1.53)	0.580		
Type of Specimen	Nasopharyngeal	135(14.2)	816(85.8)	1.0		1.0	
	Throat swab	22717.7)	1056(82.3)	1.310(1.04-1.65)	0.022^a^	1.170(.91-1.50)	0.215
Region	Addis Ababa	184(17.2)	888(82.8)	1.145(.92-1.44)	0.237		
	Other region	178(15.3)	984(84.7)	1.0		1.0	
Influenza test result	Positive	21(13.3)	137(86.7)	1.0		1.0	
	Negative	341(16.6)	1714(83.4)	1.49(0.94-2.39)	0.092		
SARS-CoV-2	Positive	15(20.3)	59(79.7)	1.0		1.0	
	Negative	34716.1)	1813(83.9)	0.75(0.42-1.34)	0.34		
Year	2021	82(24.7)	250(75.3)	1.9(1.44-2.51)	<0.001^a^	1.170(.79-1.73)	0.434
	2022	280(14.7)	1622(85.3)	1.0		1.0	
Season	Spring(Mar-May)	79(12.3)	562(87.7)	0.83(0.61-1.14)	0.25	.81(.59-1.12)	0.204
	Summer(Dec-Feb)	63(15.9)	333(84.1)	1.12(0.80-1.58)	0.50	1.12(.79-1.58)	0.516
	Autumn(Sep-Nov)	113(24.8)	342(75.2))	1.96(1.46-2.63)	<0.001	1.67(1.17-2.38)	0.005^b^
	Winter(Jun-Aug)	107(14.4)	635(85.6)	1.0		1.0	

Key: AOR, adjusted odds ratio; COR, crude odds ratio.

a Significant association in COR

b Significant association in AOR.

### Distribution of RSV across the sentinel sites and month

The RSV was detected across all sentinel sites, except the Gambella, Gode, and Shiromeda sites. The highest RSV positivity was detected in cases enrolled in the Akaki health center (29 [31%]), followed by Adare (58 [23%]) and Felegehiwot hospitals (18 [20.0%]). Nearly a quarter of the detected RSV cases were from the Yekatit 12 hospital sentinel site (91 [18.1%]) (Supplemental Figure 1).

**Figure 2:**
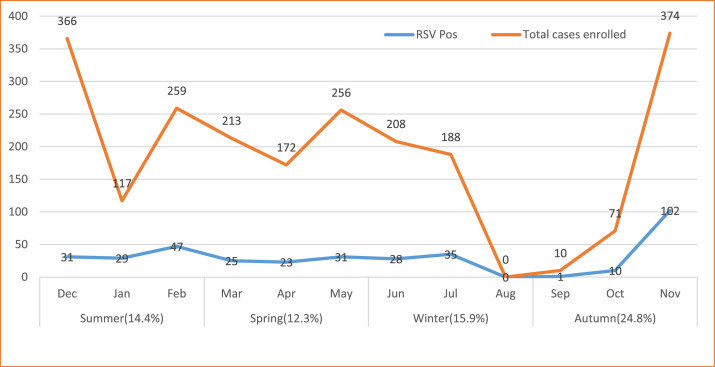
Distribution of total enrolled cases and RSV positivity by season, Ethiopia. The percent in brackets of the seasons represent the RSV positivity rate of each season. RSV, respiratory syncytial virus.

The RSV was detected in all four seasons, with highest positivity rate in fall (113 [24.8%]), followed by the rainy season of winter (63 [15.9%]). It was circulating year-round, with highest positivity rate in the month of November, followed by January, July, and February. Laboratory testing of the RSV was interrupted in the August owing to a shortage of laboratory detection kits (Figure 2 and Supplemental Figure 2).

### Factors associated with RSV infections

The age category of children, type of samples collected, and seasonality were significantly associated with RSV positivity rate in the bivariable logistic regression. However, the type of specimen collected was not significantly associated in the multivariable logistic regression. In the multivariable analysis, the age of the study participants was significantly associated with the RSV positivity rate (adjusted OR [AOR] 2.8, 95% CI 1.9-4.1) for age <1 year and (AOR 1.9, 95% CI 1.3-2.7) for the age category of 1-<2 years) compared with the age category of 3-5 years. In addition, RSV test positivity was significantly associated with the fall season (AOR 1.67 95% CI 1.17-2.38). Although, sex, residential region of cases, and case classification, influenza or SARS-CoV-2 result status had no statistically significant association in the overall RSV infection in the study participants (Table 2).

## Discussion

This study aimed to assess the prevalence of RSV in children aged under 5 years across the influenza sentinel surveillance sites in Ethiopia. The overall RSV positivity was 16.2%. The prevalence was higher in younger aged children and during the season of fall.

Our study finding revealed that RSV is one of the major contributing agents in children aged under 5 years with ARI. The 16.2% RSV positivity rate is, by far, higher than the influenza test positivity rate reports of 6.7% in 2018 [15] and 5.9% in 2020 [16] that were conducted in Ethiopia. In addition, the RSV positivity rate from our study is higher than the influenza positivity rate of 8.0% and SARS-CoV-2 (3.3%) during the same study period and setting for the same age category (unpublished influenza sentinel surveillance data from the Ethiopian Public Health Institute). This indicates that the RSV accounts for a large share of ARI in children under 5 years of age, which needs a huge emphasis in disease prevention, control, and management by concerned bodies.

The epidemiology of RSV-associated ARI has not been explored well in Ethiopia previously. In a previous study by Weldetsadik et al. [17] by enrolling 117 children from a single tertiary hospital, the positivity rate was 22.2%, which is in agreement with our age disaggregated report of a 22.8% positivity rate in children aged under 1 year [17]. Compared with studies in other countries, our overall positivity rate of 16.2% was also similar with that of the 15.5% in Senegal [18], 14% in Kenya [19], and 18% and 16.0% in China [20]. However, our study finding was lower than that of the study reports of 20.7% in Kenya [21], 40.5% [22] in Morocco, and 46.1% in Kashmir India [23]. The higher positivity rate from previous studies other than ours might be owing to a single densely populated slummy study site in Kenya and the use of combined oropharyngeal and nasopharyngeal specimen testing and a shorter date of disease onset during study participant enrollment in Morocco. The lower positivity rate of our study finding compared with the 24.4% of South Africa [24] might be owing to the study participant characteristics, which included HIV-positive cases and the majority of them were children under 1 year of age, in whom underlying health conditions and age play a role in RSV infection risk in many other studies. Moreover, the shorter date of sample collection from date disease onset and the very young age (>3 months) of study participants, the majority of whom have underlying health conditions, included in the study from India might also be a reason for the higher positivity rate than ours. Moreover, the time of COVID-19 pandemic also may play great role because the infection prevention and control practice during the pandemic and before the pandemic might be different. Our study was conducted during 2021-2022, when the infection prevention and control measures were more practiced, in line with the COVID-19 pandemic prevention and response directives/guidance. On the other hand, our finding showed a higher positivity rate than the 13.5% in Gabon [25] and 11.8% in the Philippines [26]; in the case of Gabon, this may be because the study participants included were mainly ambulatory (ILI) cases, and, in the case of the study in the Philippines, RSV test results from samples that were archived for a long time were used; both cases could contribute to the lower RSV test positivity difference.

In the present study, children below the age of 2 years were more affected than the other age group, and similarly higher RSV positivity rates for lower age groups were reported in Congo and Pakistan [27,28]. This higher positivity rate might be owing to the higher rate of nosocomial spread of the RSV virus in the pediatric groups and, of course, owing to the immature level of immunity against the RSV infection. This study also indicated that the RSV positivity rate was the highest in fall season, especially in month of November. The RSV seasonal distribution is more common in fall (24.8%), followed by the rainy season (15.9%), which revealed a similar seasonal distribution of RSV [17] and influenza virus in Ethiopia [15,16]. Reports from other countries in the tropics and subtropics presented a higher RSV test positivity during the rainy seasons [25,[28], [29], [30]. The difference may be owing to the length/season of the study, distribution of study participants across all months of seasons, and other factors.

### Strength and limitation of the study

Fairly, nationally representative, large data collected from 19 study sites in Ethiopia were used. The gold-standard and reliable RT-PCR testing was used to ensure the quality of the RSV test results. To the best of our knowledge, this is the first study finding produced from the analysis of fresh specimens collected from such a large number of study sites across Ethiopia. This study has also limitations which include the absence of clinical features of the study participants and the sample distribution was not proportionally allocated to each month of the study period, which may affect the seasonal distribution of the RSV in the study.

## Conclusion

The study revealed that the RSV positivity rate was 16.2% in children aged under 5 years in Ethiopia. The RSV positivity was highest in children under 2 years of age. The highest prevalence was observed during the season of fall, followed by the rainy season of summer. Age and seasonality were strongly associated with RSV positivity.

Because the RSV circulation in cases under the age of 5 years was considerably high, it should be considered in the prevention and control of infection and case management procedures. The data quality needs to be improved to successfully meet the surveillance objectives. Further studies on the RSV viral genotype, clinical characteristics, and additional factors, including underlying health conditions and disease outcome, need to be conducted for a better understanding of the virus and epidemiology of infection and to design a better intervention mechanism.

## Declarations of competing interest

The authors have no competing interests to declare.

## Appendix Supplementary materials

Image, application 1
