# Supplementary material for: Prevalence of respiratory syncytial virus infection and associated factors in children aged under five years with severe acute respiratory illness and influenza-like illness in Ethiopia
Source: IJID Reg. 2024 Jan 17;10:191–6. doi: 10.1016/j.ijregi.2024.01.004 (PMC10864198; doi:10.1016/j.ijregi.2024.01.004)
Supplement: Supplementary file 1 [file mmc1.docx]

**Supplemental Table 1**: List of ILI and SARI sites in Ethiopia.

| **S.N/ID** | **List of Sites** | **Type of site** | **Region/location** | **S.N/ID** | **List of Sites** | **Type of site** | **Region/location** |
| --- | --- | --- | --- | --- | --- | --- | --- |
| 1 | Yekatit 12 Hop | SARI | Addis Ababa | 12 | Butajira Hospital | SARI | SNNPR |
| 2 | Zewditu MH | SARI | Addis Ababa | 13 | Felege Hiwot Hosp | SARI | Amhara |
| 3 | St. Peter Hospital | SARI | Addis Ababa | 14 | Lalibella Hospital | SARI | Amhara |
| 4 | Akaki HC | ILI | Addis Ababa | 15 | Gambella Hosp | SARI | Gambella |
| 5 | Dilfire HC | ILI | Addis Ababa | 16 | Gode Hosp | SARI | Somali |
| 6 | Kolfe HC | ILI | Addis Ababa | 17 | Dubti Hospital | SARI | Afar |
| 7 | Shiromeda HC | ILI | Addis Ababa | 18 | Asosa Hospital | SARI | BGU |
| 8 | Adama Hosp | SARI | Oromia | 19 | Dilchora Hospital | SARI | Dire Dawa |
| 9 | Shanan Gibe Hosp | SARI | Oromia | 20 | Mekele Hospital | SARI | Tigray |
| 10 | Adare Hosp | SARI | Sidama | 21 | Jinka General Hospita | SARI | SNNPR |

Supplemental Figure 1: SARI /ILI case and RSV positive cases distribution by facility among under five children with SARI and ILI, 2021-2022

**Supplemental Figure 2**: Trends and RSV positivity among under five children SARI/ILI cases across the four seasons
